# USP9X expression is functionally related to laryngeal cancer

**DOI:** 10.7150/jca.81054

**Published:** 2023-02-27

**Authors:** Yu-feng Wan, Chen-yu Zhang, Xiao-wen Cheng, Long-sheng Liu, Tao Zhou, Jun-kang Gao, Hua-qing Zhu, Ye-hai Liu

**Affiliations:** 1 Department of Otolaryngology-Head Neck Surgery, The Affiliated Chaohu Hospital of Anhui Medical University, Hefei, Anhui, 238001, P.R. China; 2 Department of Otorhinolaryngology Head and Neck Surgery, The Second People's Hospital of Hefei, Hefei, Anhui, 230001, P.R. China.; 3 Department of Clinical Laboratory, The First Affiliated Hospital of Anhui Medical University, Hefei, Anhui, 230022, P.R. China; 4 Anhui Province Key Laboratory of Translational Cancer Research, Bengbu Medical College, Bengbu, Anhui, 233030, P.R. China; 5 Laboratory of Molecular Biology and Department of Biochemistry, Anhui Medical University, Hefei, Anhui, 230032, P.R. China; 6 Department of Otolaryngology-Head Neck Surgery, the First Affiliated Hospital of Anhui Medical University, Hefei, Anhui, 230022, P.R. China

**Keywords:** laryngeal cancer, USP9X, proliferation, migration, invasion, Mcl-1

## Abstract

An increasing number of studies have shown that USP9X is closely related to cancer. However, its role in carcinogenesis and progression of laryngeal cancer has not yet been investigated. In this study, we found that USP9X was upregulated in laryngeal cancer tissues. The expression of USP9X was significantly correlated with degree of laryngeal cancer differentiation and lymphatic metastasis. USP9X knockdown led to a decrease in the ability of proliferation, migration, and invasion of FaDu cells. The proportion of FaDu apoptotic cells increased by interfering with the endogenous expression of USP9X. We speculated that inhibiting USP9X might induce apoptosis in FaDu cells by downregulating Mcl-1 and upregulating Bax protein expression. Our findings for the first time suggest the expression level and trend of USP9X in laryngeal cancer tissue and USP9X may plays an important role in promoting the occurrence and progression of laryngeal cancer. USP9X may be a potential target for intervention in treatment of laryngeal cancer.

## Introduction

Laryngeal squamous cell cancer (LSCC) is a malignant cancer of the head and neck, and its incidence is second only to oral squamous cell cancer. The 5-year survival rate of patients with LSCC is less than 30%. In China, more than 25,000 new cases of laryngeal cancer are reported annually, and the number of cases is increasing each year 1,2. Recently, the effect of advanced laryngeal cancer treatment has been low, and recurrence rate has been high owing to the lack of a definite diagnosis 3.

Ubiquitination and deubiquitination are important processes in post-translational modification and cell cycle function 4,5. The deubiquitinating enzyme family (DUBs) can deubiquitinate substrate proteins. When DUB expression is incorrect, its involvement in the regulation of cell signal pathway transduction and the growth and development cycle are affected, causing a variety of adverse consequences including cancer, neurodegenerative diseases, and immunosuppressive disorders 6. Similar to other members of DUBs, the ubiquitin-specific peptidase 9 X-chromosome (USP9X) transforms the ubiquitination process into a deubiquitination process through a specific chemical reaction 7,8, thereby regulating cell proliferation and apoptosis 9, cell growth and migration 10, mucin polar protein formation 6, cell signal transduction pathways 11, lysosomal autophagy 12, immune inflammatory defense response 13, and catalytic metabolic response 14.

To date, USP9X has been found to be present at abnormal levels in many different tumor types including digestive tract tumors, respiratory tract tumors, hematological tumors, and genitourinary tumors. USP9X promotes or inhibits cancer 15-21 and has also been reported to be involved in the pathological processes of some chromosomal diseases, including Turner syndrome caused by sex chromosome aberrations 22, mental retardation involving the X chromosome 23, Alzheimer's disease, Parkinson's disease, and other neurodegenerative diseases 24. However, the involvement of USP9X in the formation and regulation of cancer has not been clearly studied, and the specific function of USP9X in laryngeal cancer needs to be confirmed in further studies. The purpose of this study was to comprehensively analyze the expression of USP9X, its effect on the cell cycle function, and its role in the formation and development of laryngeal cancer.

## Materials and methods

### General information

We collected 86 paraffin tissues came from 84 male and two female patients with laryngeal cancer who underwent surgery in the Department of Otolaryngology-Head Neck Surgery at the First Affiliated Hospital of Anhui Medical University from May 2019 to May 2020. Staging was based on the TNM staging plan published in the eighth edition of the International Anti-Cancer Association in 2017: 3 cases were stage I, 11 cases were stage II, 42 cases were stage III, and 30 cases were stage IV. We collected 36 fresh laryngeal cancer and adjacent normal specimens between May 2019 and May 2021 from the Department of Otolaryngology-Head Neck Surgery at Affiliated Chaohu Hospital of Anhui Medical University, from 35 males and 1 female. The staging plan was the same as that for the previous specimens: 2 case were stage I, 5 cases were stage II, 14 cases were stage III, and 15 cases were stage IV. All patients fulfilled the following inclusion criteria: (1) postoperative pathology-confirmed LSCC, (2) no previous history of other malignant tumors, (3) no other anti-tumor therapy before surgery, and (4) met the relevant ethical requirements. Immediately after sectioning, the tissue samples were frozen at -80 ℃ to prevent degradation.

The Immunohistochemistry (IHC) kit was purchased from Beijing Zhongshan Jinqiao Biotechnology Company, the PCR kit was purchased from TAKARA, laryngeal cancer FaDu cells (RRID: CVCL_1218) were purchased from Cell Bank of the Chinese Academy of Sciences, RPMI-1640 medium was purchased from BI, fetal bovine serum was purchased from Vivacell, rabbit anti-human USP9X was purchased from Abcam, anit-Bax, anit-Mcl-1, anti-β-actin monoclonal antibody, and the CCK-8 kit were purchased from Beyotime. USP9X siRNA was purchased from GenPharma, Lipofectamine 2000 was purchased from Invitrogen, the transwell chamber was purchased from Millipore, and the apoptosis detection kit Annexin V-PE/7-ADD was purchased from BD Biosciences. All experiments were performed with mycoplasma-free cells.

### IHC

According to the manufacturer's instructions, we performed dewaxing, antigen repair, inactivation of endogenous peroxidase, sealing, addition of the primary antibody, addition of the second antibody, DAB reagent color, hematoxylin re-staining, dehydration, xylene transparency, and sealing. After taking pictures, the results were interpreted using ImageJ software. The level of protein expression was evaluated based on the intensity and range of positive cells. The intensity of positive cells was divided into four grades: acellular staining (grade 0), light-yellow granules or masses (grade 1), yellow granules (grade 2), or brown granules (grade 3). The specimen was divided into five grades according to the number of positive cells: <1% (grade 0), 1-25% (grade 1), 26-50% (grade 2), 51-75% (grade 3), and 76-100% (grade 4). The positive marker score was calculated as positive cell intensity × positive cell range. The expression results were categorized as “high expression” (4-12) or “low expression” (0-3), according to the marker score.

### Cell culture

FaDu cells cryopreserved in liquid nitrogen were resuspended and cultured in RPMI-1640, 10% fetal bovine serum, and 1% penicillin-streptomycin solution in a 5% CO_2_ cell incubator at 37 °C. Cell fluid exchange, cell passage, and cell cryopreservation were performed regularly during culture.

### Cell transfection

The cells were divided into three groups according to the experimental design: blank group without transfection (blank group), negative control group transfected with control siRNA (NC siRNA group), and experimental group transfected with USP9X siRNA (USP9X siRNA group). Transfection was performed according to the instructions. mRNA expression was detected 24-72 h after transfection by RT-PCR, and protein expression was detected 48-96 h after transfection by western blot (WB) to evaluate transfection efficiency.

### Plate colony formation test

We inoculated 400 cells into each well in a six-well plate and made two holes in each group. Cells were cultured in a cell incubator for 8-10 days. The culture was terminated when visible clones appeared at the plate orifice. The clones were stained with crystal violet dye for 30 min, washed three times with double distilled water(ddH_2_O), dried, and photographed. The number of clones in each group was compared to the average value.

### Cell proliferation test (CCK-8 method)

Cells from different groups were inoculated into 96-well plates at approximately 2,000 cells/well and cultured for 24h and 48h in a fixed incubator under the same conditions. The proliferation rate of FaDu cells was measured with the Cell Counting Kit-8 assay. The absorbance at 450 nm of each sample was measured with a microplate reader (Thermo Scientific, USA) and recorded.

### Cell scratch test

Different groups of cells were cultured in six-well plates. After growing to confluence, a scratch was made in both the experimental and control plates. The cells were gently washed, cultured in serum-free medium, and photographed at 0 and 24 h. Scratch healing rate was calculated as follows: Scratch healing rate = (0 h scratch distance - 24 h scratch distance) / 0-hour scratch distance × 100.

### Cell migration experiment

We added 600 μL RPMI 1640 medium containing 10% fetal bovine serum to a 24-well plate. The transwell chamber was placed gently above the culture medium using flame-sterilized tweezers for a few minutes to moisten the chamber. Next, 200 μL of the cell suspension was gently added to the chamber. After 24 h of culture, apoptosis was detected using flow cytometry.

### Flow cytometry

Cells in different groups were washed twice or thrice with PBS, the supernatant was discarded and 100 μL 1× Annexin V Binding Buffer was added to resuspend the cell precipitate. We added 5 μL 7-AAD and 5 μL Annexin V-PE, mixed well, and incubated the solution for 20 min at room temperature in the dark. We then added 200 μL 1× Annexin V binding buffer. Immediately after matching the sample, analysis was performed with BD Accuri C6 Flow Cytometer and corresponding CellFIT software.

### RNA extraction and real-time fluorescence-based quantitative PCR (qRT-PCR)

RNA was extracted from the experimental tissues and cells, purified, rinsed, and eluted, and the quantity and purity were analyzed. Reverse transcription and PCR were performed according to the manufacturer's instructions. The primer sequences used in the experiments are listed in Table 1.

### Western blot (WB)

The experimental tissues and cells were subjected to protein extraction, and protein quantification was performed using a BCA kit. Samples were separated by 10% SDS-PAGE and transferred to the PVDF membrane (GE Healthcare, Piscataway, NJ). The membranes were blocked with 5% BSA for 2 hours at room temperature, followed by a 4ºC overnight incubation with the primary antibodies. The secondary antibodies (Zhongshanjinqiao, Beijing, CN) were incubated for 1 hour at room temperature, and the target protein bands were detected using the ECL reaction solution (Engreen, Beijing, CN).

### Statistical analysis

Data were analyzed using GraphPad Prism software (version 7.0). The measurement data for each group is expressed as mean ± standard error of the mean. Comparisons between the two groups were made using Student's t-test. When comparing multiple groups, one-way analysis of variance was used if there was only one control variable. Confounding factors for comparison of clinicopathologic baseline characteristics between groups were balanced by propensity score matching (PSM) method. Statistical significance was set at *p* < 0.05.

## Results

### Expression of USP9X were increased in laryngeal cancer tissues

**Figure 1:**
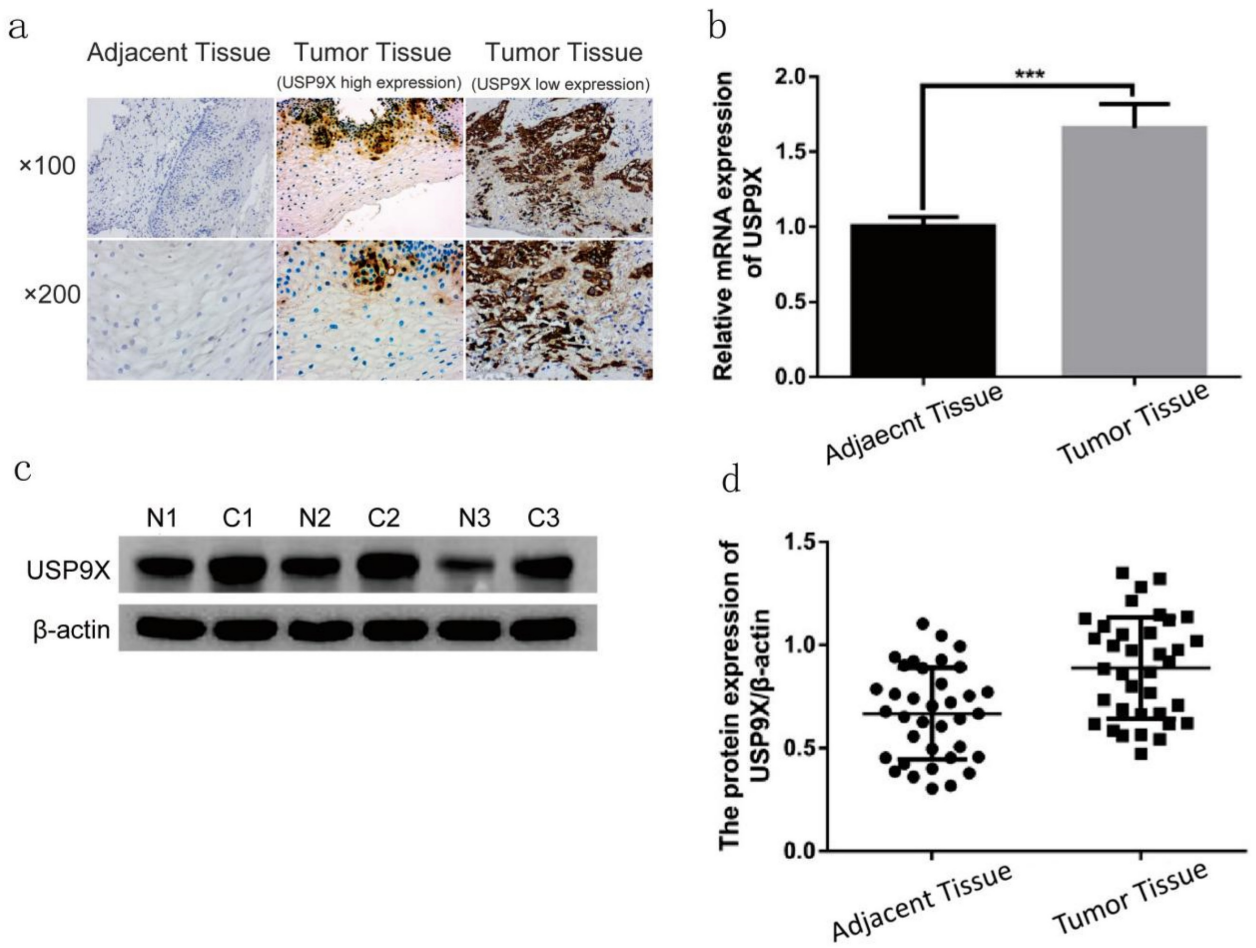
** USP9X expression was increased in laryngeal tumor tissues.** (a) USP9X expression was examined by IHC in laryngeal tumor tissues and adjacent tissues. (b) The mRNA levels of USP9X were detected by qRT-PCR in tumor tissues and adjacent tissues. ***P<0.05. (c and d) WB analysis of USP9X in laryngeal tumor tissues and adjacent tissues.

The expression of USP9X in 86 cases of laryngeal cancer tissues were examined by IHC. USP9X high expression were detected in 73 cases (84.88%), and USP9X low expression were detected in 13 cases (15.11%). USP9X was mainly expressed in the cytoplasm and was deeply and broadly stained. The expression of USP9X in 36 cases of laryngeal cancer tissues were examined by qRT-PCR and WB. QRT-PCR analysis showed that the expression of USP9X mRNA in laryngeal cancer tissues was higher than that in the adjacent tissues. The USP9X mRNA level in laryngeal cancer tissues was approximately 1.65 times that in the adjacent tissues (*p* < 0.001). WB analysis showed that USP9X protein was expressed in paraneoplastic and laryngeal cancer tissues. The expression of USP9X protein in most laryngeal cancer tissues (31/36) was higher than that in the adjacent tissues. The gray value of laryngeal cancer tissues was approximately 1.33 times that of the adjacent tissues (*p* < 0.001, Figure 1).

Further studies on the relationship between USP9X expression of 86 paraffin specimens and different clinical features of enrolled patients with laryngeal cancer showed that the increased expression of USP9X was not significantly associated with sex, age, clinical stage, or lymph node metastasis* (p* > 0.05*)*. However, this differed significantly with the degree of differentiation* (p* < 0.05*)*, indicating that abnormal expression of USP9X in laryngeal cancer was related to the parameter (Table 2). The relationship between USP9X expression in 36 fresh tissues and different clinical parameters of enrolled patients with laryngeal cancer also has been analyzed. As shown in Table 3, abnormal expression of USP9X in laryngeal cancer was closely related to gender, clinical stages, degree of differentiation and lymphatic metastasis (*p* < 0.05).

Next, PSM analysis was used to balance the confounding factors for comparison of clinicopathologic baseline characteristics between USP9X high expression group and USP9X low expression group of 86 paraffin specimens of enrolled patients. The results showed that there was no statistical significance in gender, age and clinical stages between the High expression group and the Low expression group. As for the degree of differentiation, the proportion of high differentiation in the High expression group (7/12,58.3%) was significantly higher than that in the Low expression group (7/12,50%) (*p* < 0.05). With regard to lymphatic metastasis, the proportion of lymphatic metastasis in the High expression group (12/12,100%) was significantly higher than that in the Low expression group (2/12,16.7%) (*p* < 0.05) (Table 4).

### USP9X downregulation decreased the proliferation, colony formation, and migration abilities of FaDu cells

**Figure 2:**
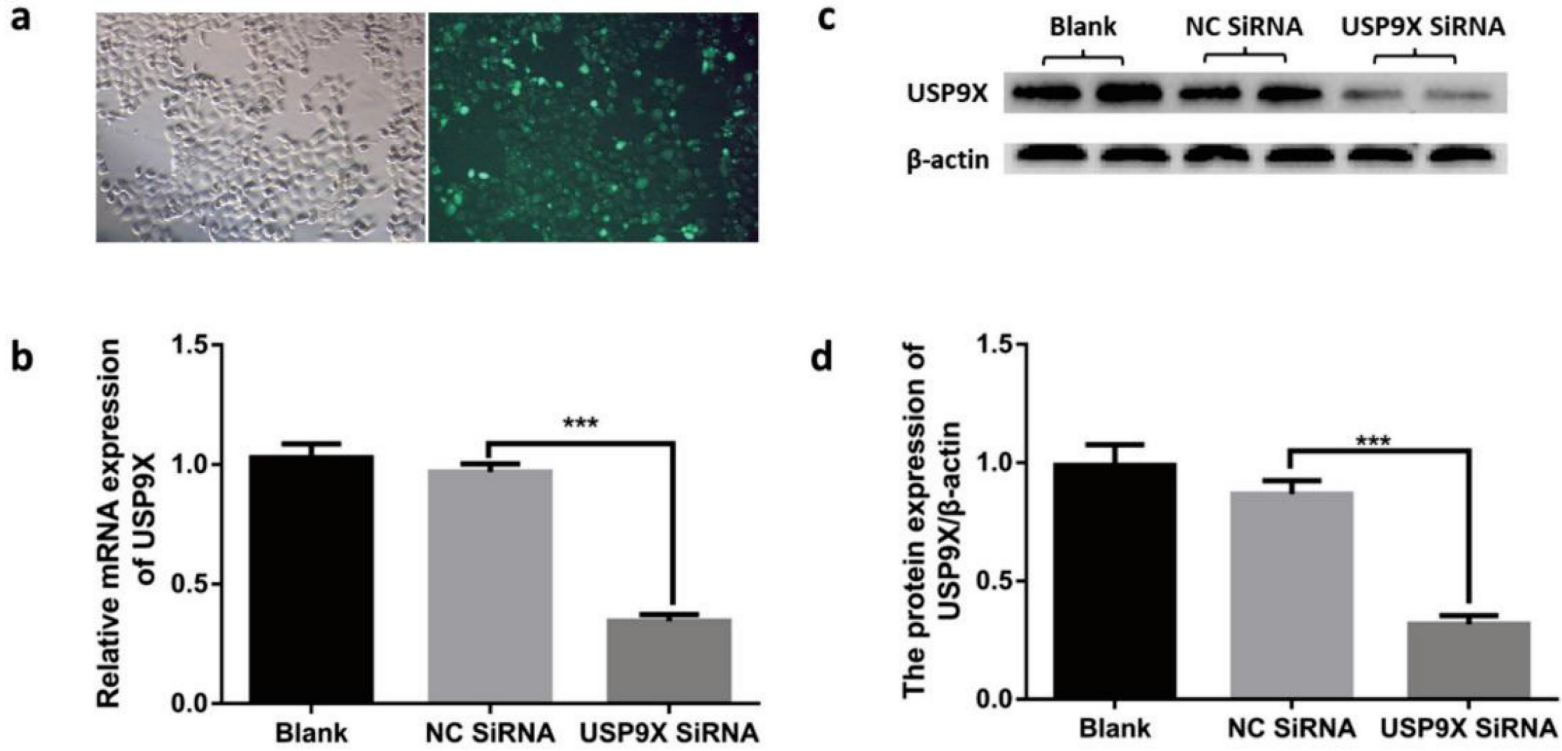
**Testing transfection efficiency in different groups.** (a) After transfection with fluorescent reagent, FaDu cells were observed for green fluorescence under a microscope. USP9X expression in FaDu cells in different groupswere detected by (b)qRT-PCR and (d) WB. (c) WB analysis was performed to measure the level of USP9X protein in different groups.

According to the transfection reagent manual, the cells were photographed under a fluorescence microscope at 12 h after transfection. Expression of 5-FAM green fluorescent dye was detected using blue light excitation. After transfection, USP9X mRNA and protein were detected at 48 h and 72 h. The results showed that the expression of USP9X mRNA and protein in siRNA-USP9X-FaDu cells in the Si-USP9X group was lower than that in the blank and NC siRNA groups (Figure 2).

**Figure 3:**
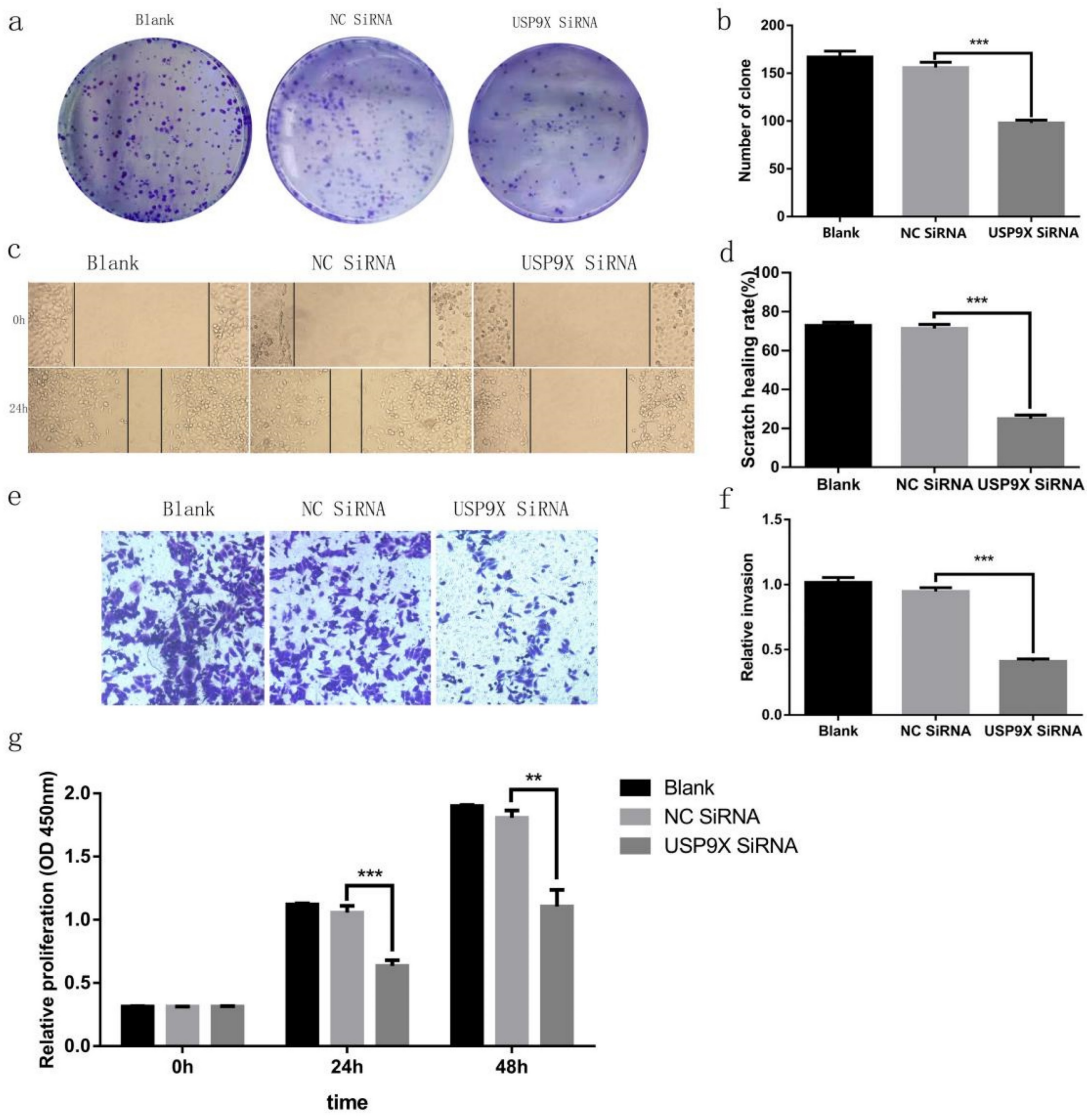
**USP9X expression affects the proliferation and migration of FaDu cells *in vitro*.** (a and b) Representative images and quantification of the colony forming assay in FaDu cells in different groups. n = 3, ***p < 0.001. (c and d) The wound healing experiment analyzed the variation of USP9X on migration ability in different groups. n = 3, ***p < 0.001. (e and f) Transwell assay was performed to determine the effect of USP9X knockdown on the migration ability of FaDu cells. n = 3, ***p < 0.001. (g)The CCK8 method was performed to measure the proliferation of FaDu cells in different groups. OD450 values were compared at the indicated time points. n = 3, **p < 0.01, ***p < 0.001.

The plate cloning test evaluated the clone-forming ability of the different groups. The experiment showed that the number of clones in the blank and NC siRNA groups was much more than that in the USP9X siRNA group (*p* < 0.001). Therefore, the clone-forming ability of FaDu cells in the blank and NC siRNA groups was stronger than that of cells in the USP9X siRNA group (Figure 3a and 3b). The CCK8 cell proliferation test showed that FaDu cells grew slowly in the USP9X siRNA group and grew more rapidly in the blank and NC siRNA groups (Figure 3g).

Cell migration ability was assessed across the different groups. The scratch healing rate at 24 h in FaDu cells in the blank group and NC siRNA control group was significantly higher than that in the USP9X siRNA group (*p* < 0.001). Therefore, the growth and migration rates of FaDu cells in the blank and NC siRNA groups were higher than those in the USP9X siRNA group (Figure 3c and 3d). The invasive ability of the different groups was detected using a transwell cell invasion assay. The results showed that the number of cells penetrating the membranes in the USP9X siRNA experimental group was significantly lower than that in the untreated blank and NC siRNA groups (*p* < 0.001). Therefore, the invasive ability of FaDu cells in the blank and NC siRNA groups was stronger than that of cells in the USP9X siRNA group (Figure 3e and 3f).

### USP9X knockdown promoted FaDu cell apoptosis

Apoptosis in the different groups was detected using flow cytometry. The results showed that the percentage of living cells in the USP9X siRNA experimental group was significantly lower than that in the blank and NC siRNA control groups (*p* < 0.01). In addition, the early apoptosis rate in the USP9X siRNA group was significantly higher than that in the untreated blank and NC siRNA groups (*p* < 0.001). Therefore, the apoptotic ability of FaDu cells in the blank and NC siRNA groups was weaker than that of cells in the USP9X siRNA group.

**Figure 4:**
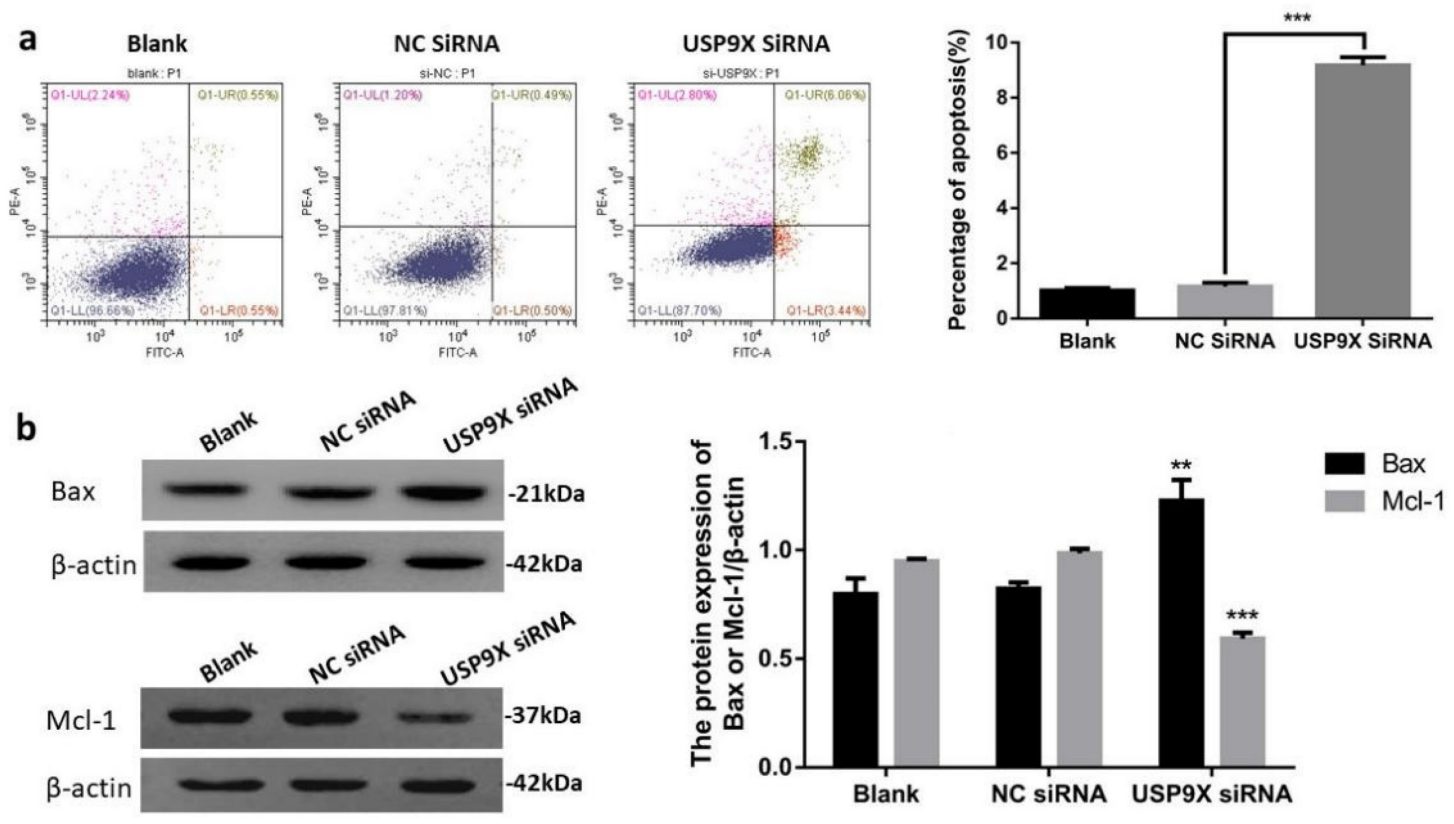
**Downregulation of USP9X expression led to increased apoptosis of FaDu cells.** (a)Flow cytometry was used to detect the effect of USP9X on apoptosis in FaDu cells. n = 3, ***p < 0.001. (b) WB detected blot analysis of Bax and Mcl-1 protein expression in different treatment groups. n = 3, **p < 0.01, ***p < 0.001.

To verify the effect of decreased USP9X expression on apoptosis-related proteins, WB was used to detect the expression of Bax and Mcl-1 proteins in different treatment groups. The results showed that the expression of Bax protein in the USP9X siRNA group was upregulated and Mcl-1 was downregulated (*p* < 0.01) compared with that in the untreated blank and NC siRNA groups. Therefore, the apoptotic ability of FaDu cells in the USP9X siRNA group was stronger than that of cells in the blank and NC siRNA groups, i.e., low USP9X expression was related to the expression of Mcl-1 and Bax (Figure 4).

## Discussion

Laryngeal cancer is a common malignant tumor in otorhinolaryngology and head and neck surgery. It accounts for approximately 2.4% of all malignant tumors in humans, with a large proportion of patients being male. Over 95% of pathological manifestations are LSCC 25, which has gradually increased in recent years. There are various causes of laryngeal cancer, but its ubiquitin-related pathogenesis is still unclear. Among the many physiological functions of ubiquitin, cell cycle is one of the most critical 26. DUBs participate in various processes of ubiquitin modification in human proteins, including recovery and processing of precursor proteins, modification of ectopic ubiquitin chains on receptor proteins, and modification of other proteins in the ubiquitin cycle 27,28. However, with progress has been made in the study of deubiquitinating enzymes in recent years, leading to breakthroughs in the development of new targets for tumor diagnosis and treatment. To date, studies have confirmed that USP9X is involved in the Hippo pathway to control apoptosis proteins Mcl-1 and YAP1 and through the fork box transcription factor FOXO3A and F-box substrate receptor protein FBXW7 to control cyclin D1 expression 16,29,30. Therefore, USP9X may play an important role in inhibiting or promoting many aspects of a tumor's executive cell signaling pathway and lymphatic metastasis.

This study was divided into two parts: 1) Study on the expression of USP9X in laryngeal tumor tissues by IHC, qRT-PCR, and WB assay; 2) Study on the changes of biological behavior of FaDu cells by using USP9X siRNA. Plate clone formation assay, CCK8 growth curve determination, cell scratch migration, transwell invasion, and flow cytometry apoptosis analysis were performed to observe the cell phenotypic changes after USP9X siRNA transfected into FaDu cells. Firstly, 86 cases paraffin specimens of laryngeal cancer were detected by IHC. The level of USP9X in laryngeal cancer was higher than that in adjacent tissues, which is consistent with the abnormal expression of USP9X in malignant tumors of other organs. Combined with the analysis of IHC results and clinical data, it was concluded that abnormal increased expression of USP9X in laryngeal cancer tissues was not correlated with sex, age, stage, or lymph node metastases, but was correlated with the degree of differentiation. QRT-PCR and WB analysis showed that there was a significant increase in USP9X mRNA transcription and protein levels in laryngeal cancer tissues, suggesting that USP9X was highly expressed. Therefore, we speculated that USP9X was positively correlated with the malignant progression of laryngeal cancer. The role of deubiquitinating enzyme USP9X in the occurrence and development of laryngeal cancer must be further studied. Secondly, the expression of USP9X in FaDu cells was inhibited using siRNA technology. QRT-PCR and WB experiments showed that expression of USP9X mRNA and protein in transfected USP9X-siRNA-FaDu cells were lower than that in the Blank and NC groups. USP9X can effectively play a promoting and containment role in different tumors. For example, one of the protooncogenic functions of USP9X stems from its interaction with the anti-apoptotic protein Mcl-1, which promotes the increased expression of Mcl-1 in cancer tissues and inhibits apoptosis 31,32. USP9X also exhibits tumor inhibitory functions by interacting with Kras 16. Therefore, USP9X is an important factor that promotes containment, and its role in the tumors depends on the background and species and its impact on laryngeal cancer must be further studied at the cellular level. To verify the effect of USP9X on the biological behavior of laryngeal cancer cells, we performed proliferation, migration, invasion, and apoptosis assays using the constructed USP9X-siRNA-FaDu cells. The results of plate clone formation assay, CCK8 assay, cell scratch migration test, transwell assay, and flow cytometry indicated that USP9X could significantly promote the proliferation and invasion of FaDu cells, in addition to inhibiting apoptosis. On this basis, we detected the expression of apoptosis-related proteins Mcl-1 and Bax in the different treatment groups by WB. The decreased expression of USP9X subsequently reduced Mcl-1 expression and increased Bax expression, which may be the cause of apoptosis in laryngeal cancer cells. The molecular mechanism of apoptosis reduction requires further study.

This study attempted to analyze the clinical significance and relationship between USP9X expression and laryngeal cancer progression. The expression rule of USP9X in laryngeal cancer tissues can provide a valuable reference for the diagnosis, treatment, and prognostic evaluation of laryngeal cancer. USP9X may be a potential intervention target for the treatment of laryngeal cancer. Future tumor therapy may involve the suppression of overexpressed USP9X and its related family of genes. Improving the stability of USP9X expression and designing a route of drug administration are key to the future application of USP9X in clinical treatment. However, the initiation and evolution of laryngeal cancer is more complex and remain unclear. Not only is the regulation of various genes by USP9X a complex and dynamic process important for the occurrence and development of cancer, there may also be more than one downstream target of USP9X. Therefore, the function and regulatory mechanism of USP9X in laryngeal cancer must be further studied.

We demonstrated that there was a clear increase in the level of USP9X in clinical laryngeal cancer tissues compared with adjacent tissues. USP9X overexpression is related to the degree of differentiation and lymphatic metastasis. USP9X knockdown led to a decrease in the ability of proliferation, migration, and invasion of FaDu cells. The proportion of FaDu apoptotic cells increased by interfering with the endogenous expression of USP9X. Inhibiting USP9X might induce apoptosis in FaDu cells by downregulating Mcl-1 and upregulating Bax protein expression. Our findings for the first time suggest the expression level and trend of USP9X in laryngeal cancer tissue and USP9X can potentially promote the occurrence and development of laryngeal cancer, providing a theoretical basis for further study of laryngeal cancer cell biology.

## Figures and Tables

**Table 1:** Primer sequences for qPCR

Gene	Forward primer	Reverse primer	Size (bp)
USP9X	CATGGACCTGGCTCTCAGTG	AAACACATGGGGACTTCGCT	220
β-Actin	AACTGGGACGACATGGAGAAAA	GGATAGCACAGCCTGGATAGCA	192

**Table 2:** Analysis of USP9X expression and clinical features in 86 laryngeal cancer paraffin specimens

Clinical features	Cases	IHC analysis of USP9X		c^2^	P
		High expression	Low expression		
Gender					
Male	84	71	13	0.664	0.415
Female	2	2	0		
Age (years)					
≤60	27	21	6	0.604	0.437
>60	59	52	7		
Clinical stages					
I-II	14	11	3	0.480	0.488
III-IV	72	62	10		
Degree of differentiation					
Poorly	31	29	2	6.448	0.040
Moderately	42	36	6		
High	13	8	5		
Lymphatic metastasis					
Yes	30	28	2	2.870	0.090
No	56	45	11		

**Table 3:** Analysis of USP9X expression and clinical parameters in 36 fresh laryngeal cancer tissues

Clinical features	Cases	IHC analysis of USP9X		c^2^	P
		High expression	Low expression		
Gender					
Male	35	30	5	53.244	0.000
Female	1	1	0		
Age (years)					
≤60	8	6	2	1.062	0.303
>60	28	25	3		
Clinical stages					
I-II	7	3	4	73.012	0.000
III-IV	29	28	1		
Degree of differentiation					
Poorly	9	6	3	59.839	0.000
Moderately	11	11	0		
High	16	14	2		
Lymphatic metastasis					
Yes	14	14	0	58.440	0.000
No	22	17	5		

**Table 4:** Comparison of baseline clinicopathological features before and after PSM matching between USP9X high expression group and USP9X low expression group

		Before matching through PSM				After matching through PSM				
Clinical features	Cases	IHC analysis of USP9X		c^2^	P		IHC analysis of USP9X			
		High	Low			Cases	High	Low	c^2^	P
Gender										
Male	84	71	13	0.664	0.415	24	12	12	--	--
Female	2	2	0			0	0	0		
Age (years)										
≤60	27	21	6	0.604	0.437	10	5	5	0.000	1.000
>60	59	52	7			14	7	7		
Clinical stages										
I-II	14	11	3	0.480	0.488	4	2	2	0.000	1.000
III-IV	72	62	10			20	10	10		
Degree of differentiation										
Poorly	31	29	2	6.448	0.040	6	5	1	7.744	0.021
Moderately	42	36	6			5	0	5		
High	13	8	5			13	7	6		
Lymphatic metastasis										
Yes	30	28	2	2.870	0.090	14	12	2	17.143	0.000
No	56	45	11			10	0	10		

## Abbreviations

LSCC: laryngeal squamous cell cancer
USP9X: the ubiquitin-specific peptidase 9 X-chromosome
IHC: immunohistochemistry
qRT-PCR: quantitative real-time polymerase chain reaction
WB: western blot
